# Dental Responsibility for Early Diagnosis of Tumors of the Mouth and Jaws

**Published:** 1891-09

**Authors:** Arthur K. Stone


### DENTAL RESPONSIBILITY FOR EARLY DIAGNOSIS OF TUMORS OF THE MOUTH AND JAWS.[1]

BY ARTHUR K. STONE, A.M., M.D.[2]

When your president asked me to write a paper for your Academy on tumors of the jaw I supposed that I had a fairly easy task before me. But after spending some time in looking over the literature of the subject I came to the conclusion that to write an article which should be of any practical value would in reality be a very difficult task. Therefore I feel that I must ask your indulgence and good nature for the shortcomings of this paper.

The first thing that strikes one who is making a study of this subject is the fact that its literature is filled with reports of mammoth tumors, unique specimens, and rare cases. These are dwelt on at great length and pictured in all their horrors, and the idea is given to the student that the terrible tumors are of frequent occurrence, while, in reality, this is not a just conclusion, for upon careful investigation of the reported cases and the articles in the "Systems of Surgery," both general and dental, the fact is developed that the pictures and the cases are repeated time and time again. In fact, many of the cases have, so to speak, become the stock in trade of writers on tumors of the jaw. And with these they have covered up the important, common, every-day facts, so that

---

[1] Read before the American Academy of Dental Science, Boston, May 6, 1891.

[2] Surgeon to the Boston Dispensary.

they can hardly be found. Even Sir Christopher Heath, in his most recent article, bases his arguments upon specimens which have been in the museums since 1834 and 1847.

It is obvious to you all that such giant tumors can have no real interest to you as dentists, or, in fact, to practitioners in general. They are interesting to us as rarities; and they are interesting to the hospital surgeon who may have them to remove, provided, of course, that he is anxious for that sort of an operation; and you and I should be pleased to see the operation, but we should feel thankful that we did not have the responsibility on our shoulders.

These large tumors are the interesting tumors, so called, but fortunately they are extremely rare. That these new growths monopolize to such an extent the literature of the subject is but a fair example of much of the medical teaching. We are taught the uncommon and rare things with the same or even more emphasis than we are taught the things of every-day occurrence. In fact, the young man in medicine has to spend a good part of his first years in practice in forgetting the freaks and learning to recognize the common, no matter under what guise it may appear. I hope that the dental teaching is much wiser in this respect.

New growths of the mouth and jaws, though common, medically speaking, are really rare. But the importance of their early observation and consequent accurate diagnosis cannot be overrated. And it is in the power of this society to do much to make severe deforming jaw operations largely unnecessary. By this I mean that in continually searching the mouths of your patients you have the best opportunity to discover new growths in their earliest stages, make an accurate diagnosis, and carry out proper treatment before the disease, if it is malignant, has gone so far that only a severe operation will suffice. Not only can you do this, but the responsibility of not doing it must rest upon you. If you will consider all tumors and ulcers about the mouth and jaws serious until an absolute diagnosis has been made, much of the terror of malignant disease of the jaw will be lost.

To accomplish this desired result, it is necessary to know—

1. What new growths are liable to be found on the lips, jaws, and tongue.

2. What the growth under consideration is.

3. The appropriate treatment.

The first requisition can come only from familiarity with the subject in the various text-books and a study of reported cases.

Second, diagnosis in doubtful cases, thanks to cocaine, can often be absolutely settled by the pathologist; while the appropriate treatment must frequently be left to the surgeon, who may need all the resources of a well-equipped hospital to enable him to treat the case in a proper manner.

A most common form of tumor of the jaw is a simple inflammatory condition due to irritation occurring about an unremoved fang of a tooth. With this as a starting-place, the pus can soon infiltrate the tissues, causing partial immobility of the jaws, on account of the large, boggy swelling of the surrounding parts, foul breath, and a general malignant appearance. Such cases are specially for the dentist to diagnose. The surgeon is apt to think too strongly of malignant tumors, and, not being so skilful in working in the small space afforded by the nearly closed jaws, easily misses the offending root and demands a radical operation. Besides, his unfavorable prognosis will unnecessarily frighten his patient. Care and patient investigation will reveal the old fang or bit of dead bone, and, when these offending causes are once removed, antiseptic applications and cleansing mouth-washes will usually quickly return the jaw to its normal state.

The ulcers of the jaws and tongue next deserve mention. They are of special importance, as in their early stages accurate diagnoses are impossible. Ulcers of the lips do not come within the scope of this paper, though it may be well to state that a really common form of ulceration of the lips—*i.e.*, cancer of the lower lip—can, by its secondary deposits, involve the jaw. But early recognition of the disease, and operation, will probably prevent this disastrous result. The operation is comparatively simple and *can* be done with cocaine.

Ulcerations in the mouth may be simple inflammatory conditions, due to the irritation caused by the sharp edge of a decayed tooth upon the tongue, cheek, or gum, or may start from wounds of the mucous membrane, caused by toothpicks or other like things held in the mouth. Sometimes the ulcerations are due to syphilis or tuberculosis, or, it may be, to malignant disease. All these ulcerations have the same general appearance at the beginning, and it is only after careful observation that one can say that a given lesion is cancer, syphilis, tuberculosis, or a simple ulcer.. In order to make the diagnosis, the position of the ulcer must be thought of. If it is where irritation from the sharp edge of a tooth or a mass of tartar can occur, the exciting cause must be removed at once and simple measures adopted to heal the ulcer. This will usually succeed, and in a few days the ulcer will disappear. But if it does not, valuable time must not be lost by making fruitless efforts to check a malignant disease. Under cocaine the surface can be scraped, or a part, a mere scrap, removed, and proper examination will probably show whether the sore is tuberculosis or cancerous.

The position of the ulcer, if it is on the tongue, may give a hint as to the diagnosis. Syphilitic gummata almost always occur on central portions of the tongue, while the cancerous and tubercular are usually on the side.

All ulcerations may have indurated bases, but epithelioma more frequently presents a hard indurated mass lying beneath the ulcer than any of the other forms. And the same is true of the neighboring lymphatic glands; they are more frequently enlarged in cancer than in the other lesions.

Mucous patches rarely occasion any great amount of trouble in diagnosis, so I will do nothing more than mention them.

In passing, it may be well to state that a man, or a woman either, can have a chancre on the lip or tongue; it is commonly near the tip of the tongue, and the induration and enlarged glands are as a general thing well marked. Try for a history, and that will settle the diagnosis. A quiet dinner, with some champagne, etc., followed by a little trip in the town, two or three nights before the appearance of the ulcer, will be pretty conclusive evidence as to the nature of the disease.

A common form of tumor seen in the mouth is the ranula. This is a cyst of one of the ducts through which the saliva of the submaxillary or the sublingual glands is conveyed to the mouth. It is caused by a stoppage in either the ducts of Wharton or of Rivini. By the plugging of these ducts the saliva is dammed back, the duct is distended, and the cyst formed. Bryant states that this is not the fact, but that the cyst is always in the mucin glands in the floor of the mouth. Examination of the reported cases shows that the so-called ranula may come from any of the above-mentioned causes. The diagnosis is easy, as the tumor is evidently cystic in character with clear, fluid contents, and careful examination will show that it is located entirely on one side of the frænum of the tongue. If there be any doubt as to the diagnosis, an exploring needle will show the contents to be clear, glairy fluid, either mucin or saliva. Sometimes simple evacuation of the contents is all that is necessary to effect a complete cure. At other times a piece must be cut out of the cyst-wall and the interior packed with gauze in order to allow it to granulate. Before passing on, it may be well to remark that a salivary calculus may be the cause of the stoppage in the duct. In such a case the presence of the stone must be determined and its removal secured before a cure can be effected.

Cysts of the jaws may be divided into three classes:

1. Simple cysts, with simply watery contents.
2. Dentigerous cysts, which in addition contain a tooth.
3. Multilocular cysts, or, as they are sometimes called, cysto-sarcomata or cysto-adenomata.

The first two varieties are benign in their growth, while the third may be either benign or malignant.

Simple cysts may spring from beneath the periosteum, and, if situated in the lower jaw, bulge the inner part of the alveolus so that they appear more prominently in the mouth than externally.

The other variety of simple cyst has its origin during the embryonic period of the tooth-follicles before the formation of the dentine and enamel. Their growth is usually slow and painless. If they attain any size so that the bone over the cyst-wall is sufficiently thinned, pressure over the tumor will give the so-called egg-shell crackle. The contents, as already mentioned, are clear and watery.

The dentigerous cysts are those which in addition to the fluid contents also contain a tooth, or the remains of a tooth, commonly one of the permanent set of teeth, though it must be borne in mind that an adventitious tooth may be the exciting cause. The crown of this misplaced tooth is usually perfect, while the roots are for the most part absorbed. To establish a diagnosis the absence of some one of the teeth is to be considered; if the bone is thin enough the crackle will be present. While the exploring needle, especially in the hand of the dentist who is skilled to distinguish between bone and enamel, will in many cases disclose the presence of the tooth at the bottom of the tumor, and the escaping fluid will show the cystic character of the growth.

The above-described cystic tumors are generally slow of growth and give but little pain. Where there is little or no deformity there is no reason for urging the removal of the tumor, as there is no danger of its ever assuming a malignant character. And, furthermore, though it may be growing comparatively rapidly for the time being, it may cease before it assumes unpleasant proportions and never give further trouble.

It must be remembered that the strange position of the growth must not make you hesitate in making a diagnosis, providing that other things point to the cystic character of the tumor. There is, for example, among the strange cases on record, one where a cystic tumor caused by an almost perfect canine tooth was found on the palatine vault.

Operation upon the cysts of the jaw consists in cutting away part of the wall of the tumor, the removal of the tooth at the bottom of the cyst if it is present, and then packing the whole cavity with gauze and allowing the cavity to fill with granulations.

The multilocular cystic tumors are of a rather different nature. They occur almost always in middle or advanced life. In younger subjects they are apt to be slow-growing, without manifesting any special tendency to spread and involve the neighboring parts; yet as the patient becomes older the tendency to malignancy is much greater. And when the growth is removed, unless great care is taken to include all the parts involved, the tumor is practically sure to return. The multilocular cyst is more frequently found in the lower than in the upper jaw. It starts as a small swelling near the socket of the tooth and slowly increases in size. Sometimes it attains large dimensions. The tumor grows within the substance of the bone, gradually expanding the compact wall, which forms a more or less complete capsule. The size of the various cysts varies from an extremely small, honey-combed condition to those which are a half-inch in diameter, or even larger. The septa between the cysts are generally ossified.

The contents of the cysts vary even in the same tumor, in some cases being clear and limpid, while in others almost gelatinous and of a dark-brown color, due to presence of blood-pigment. The histological examination shows a marked resemblance between the tumor and the enamel organ. In the tumor the alveolar walls are covered with a columnar epithelium resembling the inner layer of cells of the enamel organ. And these cells enclose a gelatinous tissue which preserves traces of a net-work of stellate cells which is comparable to the gelatinous tissue in the centre of the enamel organ. On the other hand, there are several cases where the tumor was evidently the result of an ingrowth of the epithelium of the gums. When the tumor has attained large size, some parts fluctuate and some parts crackle, while the general feel is rounded and lobulated. If the disease is located in the upper jaw it is hard to diagnose from solid tumors of the antrum, unless the cysts are unusually large. The rate of growth is very slow, and there is but little tendency to invade the surrounding parts or glands, and still more rarely do they invade the whole system. Yet when appearing late in life these tumors may manifest a marked tendency to extreme malignancy.

Under the general term epulis is understood those tumors which spring from the periosteum of the alveolus. These tumors may be either simple fibromata or sarcomata, or, very rarely, epitheliomata. Epulis means simply "on the gums;" but the growth always starts from the periosteum,—usually from the periosteum about a decayed or decaying fang. The disease is commonly seen in young adult life. The tumors may grow to large size and give much trouble from their mechanical pressure and obstruction alone. But such cases, though once apparently common, are now rare, as an early operation is as a general thing the rule and not the exception. Epulis usually makes its appearance between two teeth, looking at first much like a bit of hypertrophied gum-tissue. But before it attracts notice it has commonly passed this stage, and it is quite plainly a new growth when the patient presents himself for consultation. If the tumor is a simple fibroid it is apt to be quite tough, while if it is a sarcoma it will be probably soft and vascular. As it grows larger the teeth are separated, and there may be some neuralgic pain, though on the whole the tumor is painless in its growth.

Should the attending physician simply content himself with a removal of the growth, he will find that its return will be only a matter of a few weeks. Something more radical is demanded; how much, depends on the diagnosis. Here is a most favorable chance to make a differential diagnosis. By the use of a little cocaine, and a one-per-cent. solution is all that is required to accomplish the desired result, a piece of the tumor can be secured and sent to the pathologist, or a fresh section can be made with a good sharp razor. Examination with a low power of the microscope will probably settle the matter, as the difference between a large-celled sarcomatous tissue and a fibromata is well marked. On the result of the diagnosis depends the operation to be recommended. One or two teeth must be sacrificed at all events. But if the new growth is a fibroma, and not large, thorough scraping of the periosteal surface from which the tumor springs will usually suffice to prevent its recurrence. If, on the other hand, the tumor is a sarcoma, a goodly piece of the alveolus must come away in order to get beyond the disease. In case the very rare form of cancerous epulis should appear, a guarded prognosis must be given as to the absolute success of the operation. And at the time of the operation a much larger piece of the alveolus has to be removed, and if there is the least suspicion that the disease has invaded the jaw-bone itself, there should not be the least hesitation on the part of the operator to do a much more serious operation.

The sarcomata in general differ in their malignancy according to their cell-formation. Some have a tendency to rapidly invade the neighboring bone and tissues, while others grow slowly and are almost benign in their nature. At times they are characterized by the formation of bone and cartilage in their substance, and are then known as osteo—or chondio—sarcomata. But such fine points are of special interest to the pathologist and to the surgeon after the tumor has been removed, as thereby he is able to make a more accurate prognosis.

New growths of the antrum present so many difficulties in diagnosis and operation as to deserve a much more extended treatment than I could give in this paper, therefore I will not speak of them at all.

I have endeavored in the preceding pages simply to suggest and indicate to you the most common varieties of tumors which you may at any time run across in your practices. But as a class, tumors of the jaw are fortunately rare, and no man can expect to see enough of them to make him absolutely sure of his ground in all cases. But every one should know enough to see when it is necessary for him to shift the responsibility of any given case from his shoulders to that of a surgeon, who has all the equipment for the most severe operation which the necessity of the case may demand.

